# The Status of the Quality Control in Acupuncture-Neuroimaging Studies

**DOI:** 10.1155/2016/3685785

**Published:** 2016-05-08

**Authors:** Ke Qiu, Miaomiao Jing, Ruirui Sun, Jie Yang, Xiaoyan Liu, Zhaoxuan He, Shuai Yin, Ying Lan, Shirui Cheng, Feifei Gao, Fanrong Liang, Fang Zeng

**Affiliations:** ^1^The Acupuncture and Tuina School, The 3rd Teaching Hospital, Chengdu University of Traditional Chinese Medicine, No. 37, Shierqiao Road, Chengdu, Sichuan 610075, China; ^2^Leshan Vocational & Technical College, Leshan, Sichuan 614000, China; ^3^Dayi Chinese Medicine Hospital, Chengdu, Sichuan 611330, China

## Abstract

Using neuroimaging techniques to explore the central mechanism of acupuncture gains increasing attention, but the quality control of acupuncture-neuroimaging study remains to be improved. We searched the PubMed Database during 1995 to 2014. The original English articles with neuroimaging scan performed on human beings were included. The data involved quality control including the author, sample size, characteristics of the participant, neuroimaging technology, and acupuncture intervention were extracted and analyzed. The rigorous inclusion and exclusion criteria are important guaranty for the participants' homogeneity. The standard operation process of acupuncture and the stricter requirement for acupuncturist play significant role in quality control. More attention should be paid to the quality control in future studies to improve the reproducibility and reliability of the acupuncture-neuroimaging studies.

## 1. Introduction

Acupuncture, a traditional therapy originated from China, has been gradually accepted as an alternative and complementary therapy by the Western medical community for its undeniable efficacy for pain and chronic diseases [1–5]. As acupuncture is widely used all over the world, its underlying mechanism attracts increasing attention. Since the 1970s, several studies of acupuncture on experimental animals have proven that the integration of central nervous system (CNS) plays an important role in acupuncture efficacy [6, 7]. With the development of neuroimaging techniques such as functional Magnetic Resonance Imaging (fMRI), Positron Emission Tomography (PET), Single-Photon Emission Computed Tomography (SPECT), electroencephalography (EEG), and magnetoencephalography (MEG), using these techniques to investigate the cerebral responses to acupuncture stimulations in vivo [8] has gradually become a spotlight in acupuncture mechanism research. Over the past two decades, there are around 200 original articles having been published in English, and a growing body of evidence demonstrates the involvement of CNS in acupuncture mechanism [9]. However, it was found that the results of acupuncture-neuroimaging studies were untenable. For example, some studies on acupoint specificity showed that the cerebral responses to acupoint stimulation significantly differed from acupoint to sham acupoint [10–13]. Some studies demonstrated that there was no significant difference in cerebral reaction between acupoint and sham acupoint [14–16]. Some investigators held that the significant difference in cerebral responses between acupoint and sham acupoint was only found during* Deqi* (needle sensation) state [17]. Methodology issues might contribute to the conflict results.

As we know, design and quality control are key points which may affect the result of a study, and strict quality control plays an essential role in the guarantee of a high repeatability, especially in acupuncture-neuroimaging study for relative smaller sample size, complexity of cerebral function, and integrity of acupuncture effect. So this study aims to investigate the current status of the quality control in acupuncture-neuroimaging studies from sample size, subjects screening, manipulation procedure, and scanning mode by analyzing the original articles published in English in the latest two decades, so as to provide ideas for the development of quality control criteria in future acupuncture-neuroimaging study.

## 2. Methods

### 2.1. Searching Strategy

We searched the original articles published during 1995 to 2014 on PubMed (http://www.ncbi.nlm.nih.gov/pubmed/) using the following MeSH terms and search strategies: ((“Acupuncture” [Mesh] OR “Acupuncture Therapy” [Mesh] OR “Acupuncture, Ear” [Mesh] OR “Acupuncture Points” [Mesh] OR “Acupuncture Analgesia” [Mesh])) AND ((“Neuroimaging” [Mesh] OR “Functional Neuroimaging” [Mesh] OR “Functional MRI” [Mesh] OR “PET” [Mesh] OR “EEG” [Mesh])).

We screened the bibliographies of identified trials and reviewed articles for further potentially relevant publication. Subsequently, we screened the full texts and assessed whether these articles met the inclusion criteria.

### 2.2. Inclusion and Exclusion Criteria

The articles would be included if they were (1) original articles; (2) acupuncture-neuroimaging studies on human beings; (3) published in English; and (4) published during 1995 to 2014.

The articles would be excluded if they were (1) reviews or editorials or trial protocols; (2) acupuncture-neuroimaging studies on animals; or (3) duplicate articles.

### 2.3. Data Extraction and Analysis

We extracted the data including the author (nationality, affiliation, and component), sample size, characteristics of the participant (patients or the health, age, gender, race, handedness, emotional state, acupuncture experience, and accompanying symptoms), neuroimaging technology, acupuncture intervention (method of intervention, manipulation procedure,* Deqi*/needle sensation, and acupuncturist), and ethical review. The data analysis was conducted after data extraction.

## 3. Results

168 [7, 10–176] original articles were included in this study.

**Figure 1:**
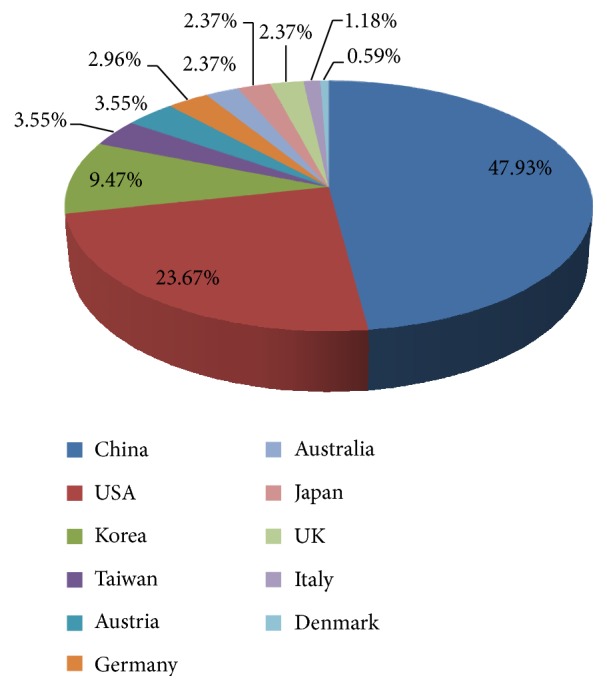
The nationality distribution of acupuncture-neuroimaging studies.

Most of the studies were conducted in China (80 studies) [10, 11, 13, 14, 16, 18–24, 26, 27, 29–31, 33–36, 43–46, 48, 51, 53, 54, 56, 58–66, 68–70, 72–75, 77, 78, 83, 84, 86, 87, 89, 90, 93, 95, 97, 99–101, 104, 111, 113, 115, 123, 137, 138, 142, 144–147, 154, 159, 161–166] and in USA (40 studies) [15, 28, 37, 38, 42, 57, 70, 71, 76, 80, 88, 91, 94, 96, 105–107, 118–120, 124–127, 129–131, 133–136, 140, 141, 148, 151, 152, 155, 156, 158, 174]. The investigators in Korea (16 studies) [7, 47, 49, 52, 55, 82, 85, 98, 102, 109, 112, 114, 116, 117, 122, 153], Taiwan (6 studies) [79, 110, 128, 170, 171, 175], Austria (6 studies) [132, 139, 150, 160, 168, 176], Germany (5 studies) [32, 50, 157, 167, 169], Australia (4 studies) [40, 41, 92, 121], Japan (4 studies) [39, 108, 149, 173], UK (4 studies) [17, 67, 81, 103], Italy (2 studies) [25, 172], and Denmark (1 study) [143] also published articles on acupuncture-neuroimaging (Figure 1). 60 studies [16, 19, 23, 25, 26, 37, 38, 42, 45, 46, 55, 56, 60, 62, 65, 67, 70–72, 75, 78, 81, 87, 88, 90, 93, 97, 100, 101, 104, 106, 107, 110, 111, 113–115, 117–121, 123, 126–129, 132, 134, 137, 142, 145–147, 150, 157, 160, 162, 168, 175] were performed with the cooperation of more than two countries.

### 3.1. Sample Size

The average sample size of these studies was 15 participants per group. For the studies performed on patients, the average sample size was 16 participants per group, while the maximal and minimal sample sizes per group were 55 participants and 1 participant, respectively. For those performed on healthy subjects, the average sample size was 14 per group, and the maximal and minimal sample sizes per group were 48 participants and 1 participant, respectively.

### 3.2. The Status of Participants

#### 3.2.1. Classification of Participants

122 studies [7, 11–13, 15–17, 24, 25, 30–33, 35, 36, 39, 41–45, 50, 53–57, 63, 64, 66–70, 72–76, 78–83, 85–100, 102–104, 106, 107, 109–111, 113–130, 132, 135, 136, 140, 143, 144, 147–176] were performed on healthy subjects. 25 studies were [10, 26–29, 34, 38, 49, 51, 58–60, 62, 65, 71, 84, 101, 105, 108, 112, 134, 137, 139, 142, 145] performed on patients. 21 studies [14, 18–23, 37, 40, 46–48, 52, 61, 77, 131, 133, 138, 141, 146, 165] recruited both healthy subjects and patients. 25 kinds of diseases were involved in these studies (Table 1). 19 studies [10, 14, 18, 19, 29, 34, 35, 40, 46, 51, 52, 59, 62, 65, 77, 84, 112, 134, 146] classified the subtypes of diseases.

#### 3.2.2. Age

100 studies [7, 10, 16–19, 23, 24, 26, 27, 30, 34, 35, 39–42, 44–48, 51–54, 57, 59, 60, 62, 63, 65, 69–74, 76, 77, 80–82, 84, 85, 87, 89, 92, 97, 98, 101–103, 105, 107–109, 112, 114, 116, 117, 120–122, 124, 125, 129, 131–135, 137, 139, 141–145, 148–153, 157, 158, 160, 162, 164, 166–168, 171–176] described the age range of participants. For the healthy subjects, the average age range was 18.3 years old, and the maximal age range was 62, while the minimal age range was 3. For the patients, the average age range was 29 years old, and the maximal age range was 57, while the minimal age range was 6. Taking studies on the stroke for instance, the maximal age range of the stroke patients was 52, while the minimal age range was 7. Furthermore, 66 studies [11, 13–15, 20–22, 25, 28, 29, 31–33, 36–38, 43, 49, 50, 55, 56, 58, 61, 64, 66–68, 75, 78, 79, 83, 86, 88, 90, 91, 93, 95, 96, 99, 100, 104, 106, 110, 111, 113, 115, 118, 119, 123, 126–128, 130, 136, 138, 140, 146, 147, 154–156, 159, 161, 163, 169, 176] described the average age of participants. Three studies [94, 163, 170] did not mention the age of participants.

#### 3.2.3. Gender

159 studies [7, 10–26, 28–79, 81–93, 95–114, 116–123, 125–131, 133–151, 153–169, 172–176] described the gender of the participants (56.7% male and 43.3% female). Nine studies [27, 80, 94, 115, 124, 132, 152, 170, 171] did not mention the gender of the participants.

#### 3.2.4. Race and Handedness

14 studies [66, 75, 87, 90, 99, 105, 115, 126, 135, 148, 151, 164, 167, 174] described and restricted the race of participants. 136 [10–24, 26, 28, 30–36, 39–56, 58, 59, 61–80, 83–87, 89–93, 95, 96, 98–100, 103–107, 109–111, 113–115, 117–131, 133, 135–138, 140–142, 144, 146–149, 151–157, 159, 162–164, 166, 168, 169, 171, 173–175] studies asked for the right-hand participants in inclusion criteria.

#### 3.2.5. Emotional State

The psychological assessment on the participants was performed in 4 studies [21, 31, 46, 92, 93]. The self-rating depression scale (SDS) and the self-rating anxiety scale (SAS) were used in 2 studies [31, 46]. The Beck Depression Inventory (BDI) [92] and the State Trait Anxiety Inventory (STAI) [93] were used in 1 study, respectively. Nine studies [19–22, 24, 65, 76, 92, 146] have excluded the participants with claustrophobia.

#### 3.2.6. Accompanying Symptoms

38 studies [11, 13, 15, 21, 31, 36, 42, 43, 46, 54, 57, 64, 65, 68, 73, 76, 78, 81, 87, 93, 100, 102, 104, 106, 110–121, 123, 128, 133, 135, 141, 148, 151, 153, 156, 158, 165, 174] excluded the participants with head trauma, and some studies [24, 46, 54, 73, 100, 121, 122] excluded the participants suffering from pain (including headache and dysmenorrhea).

#### 3.2.7. Acupuncture Experience

81 articles [13–16, 18, 22, 24, 26, 28, 29, 31, 33–35, 37, 38, 42, 51, 54–59, 63, 64, 67, 68, 70, 71, 73, 75, 78, 83, 85–87, 89, 90, 92, 93, 95, 96, 99, 101, 103–106, 108, 111, 113–115, 117–121, 123, 127, 130, 135, 138, 140, 144, 147, 148, 151–153, 156–158, 162, 166–169, 174] described the acupuncture experience of participants. Among these articles, 73 articles [12–16, 18, 22, 28, 29, 31, 33–35, 37, 38, 42, 51, 55, 57–59, 63, 64, 67, 68, 70, 71, 73, 75, 78, 83, 85–87, 89, 90, 93, 95, 96, 99, 101, 103–106, 108, 111, 113–115, 117–121, 123, 127, 130, 135, 138, 140, 144, 147, 148, 151–153, 157, 158, 166–169] described the participants as acupuncture naive.

### 3.3. Neuroimaging Technology

**Figure 2:**
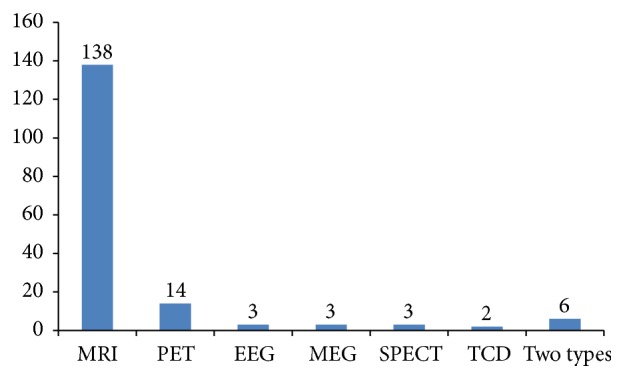
The techniques used in acupuncture-neuroimaging studies. MRI: Magnetic Resonance Imaging, PET: Positron Emission Tomography, EEG: electroencephalography, MEG: magnetoencephalography, SPECT: Single-Photon Emission Computed Tomography, and TCD: Transcranial Doppler.

137 studies [7, 11, 12, 14–26, 28, 29, 31–42, 44–50, 52, 53, 55–61, 63, 64, 66, 68–79, 84–87, 89–101, 103, 104, 106, 107, 109–115, 117–120, 122, 123, 125–129, 131–136, 138–141, 144, 146–159, 161, 162, 164, 166–168, 170, 171, 173–175] used fMRI (82.14%) to investigate the cerebral responses to acupuncture stimulation. Six studies [43, 80, 88, 116, 130, 160] used the combination of two imaging technologies. The application of the techniques in acupuncture-neuroimaging studies was shown in Figure 2.

### 3.4. Acupuncture Intervention

#### 3.4.1. Method of Intervention

**Figure 3:**
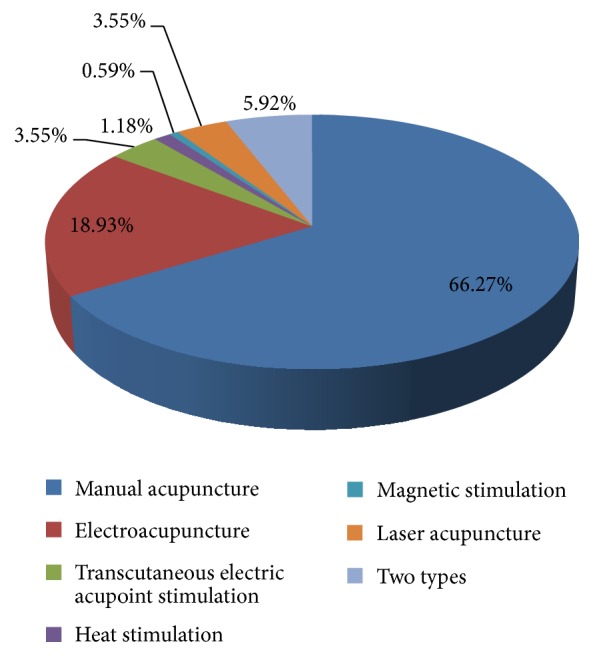
The acupuncture methods used in acupuncture-neuroimaging studies.

111 studies [7, 10, 12–14, 16–26, 28, 29, 31–34, 36, 39, 42, 43, 46–59, 61, 63, 64, 66–68, 75, 78, 82, 83, 85–88, 90, 91, 93–95, 97–100, 102–106, 108–114, 125–129, 132, 135–139, 142, 144, 145, 148, 149, 151, 153, 154, 156, 157, 163–165, 167, 172, 174–176] chose manual acupuncture as intervention method. 32 studies [27, 37, 38, 44, 60, 62, 65, 69, 70, 72, 76, 77, 80, 84, 89, 96, 101, 107, 115–117, 124, 140, 146, 152, 159, 162, 166, 169–171, 173] chose electroacupuncture as intervention method. Besides, the transcutaneous electric acupoint stimulation was performed in 6 studies [45, 73, 74, 81, 141, 143], the laser acupuncture in 6 studies [40, 79, 92, 150, 160, 168], heat stimulation on acupoints in 2 studies [147, 155], and the magnetic stimulation on acupoints in 1 study [30]. There were 10 studies [11, 15, 35, 41, 71, 131, 133, 134, 158, 161] using at least two types of acupuncture methods (Figure 3).

#### 3.4.2. Manipulation Procedure

134 articles [10–20, 22, 24, 26, 27, 29, 30, 34–39, 41, 42, 46–52, 54–70, 72, 73, 75–130, 132, 133, 135–138, 142, 144–146, 148, 151–154, 156–158, 163, 167–170, 172, 174–176] have described the manipulation procedure of acupuncture.

#### 3.4.3. *Deqi* (Needle Sensation)

82 studies [10, 15, 16, 22, 23, 26, 28, 29, 34, 35, 37, 38, 41–47, 50, 54, 56, 57, 64, 68, 69, 72, 73, 75–77, 80, 87, 89–91, 93–96, 98–101, 103, 105–111, 114, 115, 117, 118, 120–124, 127, 130, 133, 135–138, 140, 144, 146, 148, 153, 157, 158, 166–168, 172, 174–176] required* Deqi* (needle sensation) during acupuncture stimulation.

**Figure 4:**
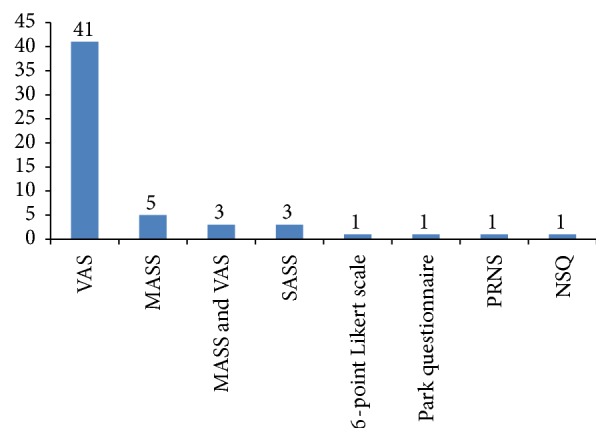
The scales/questionnaire used in needle sensation evaluation. VAS: 10-point Visual Analogue Scale, MASS: Massachusetts General Hospital Acupuncture Sensation Scale, SASS: Subject Acupuncture Sensation Scale, PRNS: Psychophysical Rating of Needling Sensation, and NSQ: Needle Sensation Questionnaire.

56 studies [10, 14–18, 28, 35, 38, 42, 43, 50, 53, 55, 57, 58, 61, 64, 66, 68, 69, 71, 73, 75–77, 87, 89–91, 93–96, 99–101, 103, 104, 106–111, 115, 117, 120, 124, 127–130, 140, 166, 169] have evaluated needle sensation after acupuncture stimulation. The 10-point Visual Analogue Scale (VAS), the Massachusetts General Hospital Acupuncture Sensation Scale (MASS), the Subject Acupuncture Sensation Scale (SASS), the 6-point Likert scale, the Park questionnaire, the Psychophysical Rating of Needling Sensation, and the Needle Sensation Questionnaire (NSQ) were used to evaluate the needle sensation (Figure 4).

#### 3.4.4. Qualification of Acupuncturists

99 articles [12, 14–19, 22–24, 26, 28, 29, 31–42, 44, 46–50, 52, 54–61, 63, 64, 66, 67, 69, 71, 72, 75, 78, 80, 82–84, 86, 87, 89–91, 93–98, 100–104, 106, 108–112, 114–117, 119–124, 126–129, 133, 135, 139, 140, 142, 144, 150, 158, 166, 174] have mentioned the qualification of acupuncturists.

### 3.5. The Ethical Review

139 studies [10–18, 20–29, 31–36, 38–45, 47–58, 60, 63–79, 81–86, 88, 90–93, 96–107, 109–115, 117–121, 123–125, 127, 128, 130–133, 135, 136, 138–148, 150–156, 158–162, 164, 166–168, 172, 174, 175] have mentioned the ethical review in the study.

## 4. Discussion

Owning to the complexity of the brain function and the diversity of acupuncture manipulations, the different or even reversed results occurring in similar acupuncture-neuroimaging studies become a common phenomenon. Seeking reasonable and practical methods is essential to improve the reproducibility and reliability of results in acupuncture-neuroimaging studies. Based on the open published literatures, this study analyzed the status of quality control in acupuncture-neuroimaging study for the first time and tried to provide some new ideas for future studies.

### 4.1. Sample Size

The appropriate sample size is important for designing an acupuncture-neuroimaging study. Bigger sample size increases statistical power because the standard error of the mean decreases by the square root of number (*N*). Due to the potential radioactivity (PET/SPECT) and the costs of imaging, the sample size in most of the neuroimaging studies was small. Some investigators suggested that 12 to 15 subjects per group could get statistical power in fMRI studies [177, 178]. Others held that the method should ensure large sample size to use rigorous corrections for multiple tests [179]. In this study, we found that the average sample size was 15 participants per group, and the average sample size for patients was slightly bigger than that for healthy subjects (16 versus 14 per group). Nowadays, most investigators agreed that, to achieve the stable statistical power, bigger sample size (at least 20 participants per group) was needed in the future acupuncture-neuroimaging study [180].

### 4.2. The Selection of Participants

The rigorous inclusion and exclusion criteria, as important guaranty for the homogeneity of the participants, are of great significance in the quality control of clinical trial.

#### 4.2.1. Classification of Participants

This study indicated that the majority of neuroimaging studies (72.78%) were performed on healthy subjects. It might be a reason for the inconsistent results. Because the traditional Chinese acupuncture theory holds that acupuncture treatment focuses on strengthening the body resistance to removing pathogenic factors and restoring the balance of* Yin* and* Yang*, the efficacy of acupuncture treatment is specific to the pathological conditions (imbalance of* Yin* and* Yang*), not the physiological state (*Yin* and* Yang* in equilibrium). So during the pathological conditions, the actions of acupoints are disease-oriented, while in the physiological state, the acupoint keeps in silence and the actions of acupoints lack orientation. In this case, patient is the better choice for acupuncture-neuroimaging studies.

In this study, we found that there was a preponderance of nervous system disorders such as stroke among the diseases involved in acupuncture-neuroimaging studies. The result indicated that acupuncture stimulation promoted the action of neural rehabilitation and its mechanism is a focus of acupuncture study. Furthermore, we noticed that some studies were performed on functional disorders such as functional dyspepsia and irritable bowel syndrome [65, 101]. As we know, regulating functional disorder is the advantage of acupuncture, so functional disorder might be a new approach in future studies.

Moreover, the subtypes of a disease should be taken into consideration when you choose patients as the participants in neuroimaging study for the patients with different subtypes might have functional or/and structural differences in brain. For example, schizophrenic subjects with predominantly negative symptoms have greater metabolic abnormalities than subjects with predominantly positive symptoms [181]. So, in acupuncture-neuroimaging study, it is better to choose the same subtype of a disease to ensure the homogeneity of participants.

#### 4.2.2. Demographic Characteristics of Participants

Some demographic characteristics of participants including age, gender, race, and handedness should be defined in the inclusion criteria.

The changes of cerebral function and structure come with age. Some studies indicated that the cerebral glucose metabolism decreased unevenly and brain tissues began aging after 40 years old [182, 183]. Older age directly correlated with reduced gray matter volume in bilateral rostral and right dorsal ACC [184]. So the age range of participants should not be ignored in neuroimaging studies. However we found that most of these studies (101 studies) described the age range of participants, that the average age range of healthy subjects was 18.3 years old, and that the average age range of patients was 29 years old. Even in some studies, the age range was more than 50 years old. It is better to keep the age range within 20 years to reduce the effect of outlier.

The functional and structural differences in human brain induced by handedness have long been investigated [185–188], although the mechanism remains unclear. So the majority of the current studies (137 studies) choose right-hand participants.

Furthermore, gender differences of the human brain are an important issue in neuroimaging studies. It has been identified that gender has significant influence on the regional neuronal activity [189, 190] and brain structure [191]. Race differences may lead to differences of brain function and structure. For example, it is reported that brain size varies by race [192]. In this study, we found that 14 studies (8.33%) described and restricted the race of the participants. Taking gender and race as covariates is needed when designing acupuncture-neuroimaging experiments.

#### 4.2.3. Emotional State

The psychological factors have significant influence on the function and structure of human brain. For example, Drevets et al. have found an area of abnormally decreased activity in the prefrontal cortex ventral to the genu of the corpus callosum in both unipolar depressives and bipolar depressives [193]. Our study shows that only 4 articles described the psychological assessment performed on the participants; more attention should be paid to the emotional state of subjects during the inclusion and data analysis in future studies, except for the study which focuses on the mechanism of acupuncture treating for psychological disorders.

Furthermore, our study demonstrated that participants with claustrophobia have been excluded in 9 studies. Claustrophobia is a phobic disorder which will cause panic, fear, or anxiety in the confined space. Scanning cannot be accomplished when it is performed on a participant with claustrophobia. So the participants with claustrophobia should be excluded in acupuncture-neuroimaging studies.

#### 4.2.4. Menstrual Period

In this study, we found that female participants were involved in 145 studies. Recently, some studies performed on healthy subjects indicated the cerebral functional and structural changes in menstrual period. For example, Veldhuijzen et al. [194] found that the pain-related cerebral activation varied significantly across the menstrual cycle. Hagemann et al. [195] found a significant gray matter volume peak and cerebral spinal fluid loss at the time of ovulation in females. So, for female participants, scanning should be performed during the same physiological period to avoid the possible changes in brain size and activity in menstrual cycles.

#### 4.2.5. Accompanying Symptoms/Disorders

The accompanying symptoms/disorders such as head trauma, pain (including headache and dysmenorrhea), and insomnia should be excluded as possible as we could considering their influence on the neuroimaging data. Tu et al. [196, 197] found that abnormal gray matter volume changes are presented in primary dysmenorrhea patients even in the absence of pain. Furthermore, some investigators held that blood coagulation disorders should be excluded in acupuncture studies [6].

#### 4.2.6. Acupuncture Experience

Some studies have reported the significant differences in cerebral response between the participant with acupuncture experience and the participant without acupuncture experience [198]. In our study, we found that participants in 44.04% of the studies were acupuncture naive. To ensure the consistency of the baseline of the participants and the comparability of consequence, the acupuncture experience of participants should be taken into consideration.

### 4.3. Image Technology

Among the neuroimaging technologies, fMRI (82.14%) was most commonly used in the acupuncture-neuroimaging studies. But we also found that multimodel imaging techniques became a new trend in acupuncture-neuroimaging studies for their significant advantages in improving spatial/temporal resolutions and lowing noise.

### 4.4. Acupuncture Intervention

#### 4.4.1. Method of Intervention

We found that, during 1995 to 2014, 66.27% studies have used manual acupuncture as intervention method, and 18.93% studies have used the electroacupuncture as intervention method. Among those manual acupuncture studies, 78.57% studies have described the needle manipulation. During 2005–2014, the majority of acupuncture-neuroimaging studies (68.71%) still used manual acupuncture as intervention method, and 17.01% studies used the electroacupuncture as intervention method. Among the manual acupuncture studies, 79.21% studies have described the needle manipulation. The results indicated that (1) although the stimulation of manual acupuncture is hard to be quantified for the individual differences of manipulation induced by different practitioners, manual acupuncture, as the traditional acupuncture intervention, is easier to be accepted by investigators and (2) the majority of these studies with manual acupuncture treatment describe the acupuncture manipulation including sterilization, the angle and depth of needle insertion, and the duration of retaining needle to ensure consistency of acupuncture treatment. Considering the influence of different manipulation results, we should not allocate too many acupuncturists in one neuroimaging study. In order to ensure the accuracy of the results, it is better to perform acupuncture manipulation with one acupuncturist.

Meanwhile, transcutaneous electric acupoint stimulation, magnetic stimulation, heat stimulation, and laser acupuncture were used in some neuroimaging studies. The results indicated that not only manual acupuncture and electroacupuncture, but also other acupuncture-related interventions attract increasing interests of investigators. In the future, more attention should be paid to those acupuncture methods which have not or seldom been touched such as ear acupuncture, abdominal acupuncture, and wrist and ankle acupuncture.

#### 4.4.2. Qualification of Acupuncturist and Operation Procedure

The qualification of acupuncturist and operation procedure is important in quality control of acupuncture trial. In our study, 59.5% of the studies have mentioned the qualification of acupuncturists and 79.88% of the studies have described the manipulation procedure of acupuncture. For the defined influence of the qualification of acupuncturist and manipulation procedure on clinical efficacy, the needling details including numbers of needle, depth of insertion, elicited response, and needle retention time and the background of practitioners including the duration of relevant training, length of clinical experience, and details of expertise in treating the specific condition being evaluated as well as any other experience that may be relevant to the trial should be reported according to the standards for reporting interventions in controlled trials of acupuncture (The STRICTA Recommendations) [199].

#### 4.4.3. *Deqi* (Needle Sensation) and Evaluation of Sensation

*Deqi* (needle sensation) plays an important role in acupuncture efficacy. Clinical trials have demonstrated that acupuncture with needle sensation was superior to acupuncture without needle sensation for analgesia [200] and paralysis [201]. A neuroimaging study [54] also showed the significant differences of cerebral responses under the* Deqi* and non-*Deqi* condition. So it is important to record the* Deqi sensation* in acupuncture studies.

In this study, we found that the questionnaire-based forms such as 10-point VAS, MASS, SASS, Park questionnaire, Psychophysical Rating of Needling Sensation, and NSQ were used to assess the needle sensation in acupuncture-neuroimaging studies. Among them, 10-point VAS was the most commonly used (68.3%). However, the liabilities and validities of some specific or nonspecific questionnaires/scales for needle sensation need further investigation.

### 4.5. The Author Nationality and Ethical Review

In this study, we found that 63.7% of the studies were accomplished by the cooperation of more than two countries. The international cooperation improved the study level and quality control in some degree. To get more international recognition and perfecting research, the acupuncture-neuroimaging studies need more international cooperation. Furthermore, ethics has got growing attention by the researchers. The interests of the participants should be taken in the first place. The ethics was an essential component which should be considered during the whole acupuncture-neuroimaging studies.

In conclusion, to improve the reproducibility and reliability of the acupuncture-neuroimaging studies, more attention should be paid to the quality control including sample size, participants screening, and acupuncture manipulations in future studies. A practical and standard quality control criterion should be developed to improve the acupuncture-neuroimaging studies.

## Figures and Tables

**Table 1:** The diseases involved in acupuncture-neuroimaging studies.

Category	Disease	Number of studies
Neurology	Stroke	12 studies
	Alzheimer's disease	2 studies
	Bell's palsy	2 studies
	Mild cognitive impairment	2 studies
	Parkinson's disease	2 studies
	Vascular aphasia	1 study
	Carpal tunnel syndrome	5 studies
	Migraine	2 studies

Pain	Chronic low back pain	1 study
	Fibromyalgia	1 study
	Chronic knee osteoarthritis pain	1 study
	Musculoskeletal disease	1 study

Psychonosology	Depression	2 studies
	Heavy smoker	1 study
	Heroin addicts	1 study

Gastroenterology	Functional diarrhea	1 study
	Irritable bowel syndrome—diarrhea	2 studies
	Functional dyspepsia	1 study

Pediatrics	Childhood autism	1 study
	Children with visual disorder	1 study
	Children with a severe type of cerebral palsy	1 study

Rheumatology	Rheumatoid arthritis	1 study

Dermatology	Atopic dermatitis	1 study

Myopia	Myopia	1 study
